# Correlation Between Serum Markers and Midluteal Phase Doppler Assessment of Uterine Arterial Blood Flow in Unexplained Recurrent Pregnancy Loss

**DOI:** 10.1007/s43032-024-01704-7

**Published:** 2024-10-01

**Authors:** Houqing Pang, Zhun Xiao, Zhongying Huang, Ouchan Hu

**Affiliations:** 1 Reproductive Medical Center, Department of Obstetrics and Gynecology, West China Second University Hospital, Sichuan University, Chengdu, China; 2 Key Laboratory of Birth Defects and Related Diseases of Women and Children, Ministry of Education, West China Second University Hospital, Sichuan University, Chengdu, China; 3 Department of Ultrasonic Medicine, West China Second University Hospital, Sichuan University, Chengdu, China; 4 West China School of Medicine, Sichuan University, Chengdu, China

**Keywords:** Recurrent early pregnancy loss, Doppler, Ultrasonography, Uterine artery, Anticardiolipin antibody, D-dimer

## Abstract

This study aimed to determine changes in uterine artery Doppler parameters in unexplained recurrent pregnancy loss (URPL) and to explore serum markers possibly associated with them. This retrospective case–control study included 107 URPL women and 107 control women. The mean pulsatility index (PI), resistive index (RI), and systolic-to-diastolic values for uterine arteries in URPL women were significantly higher than those in the controls (*P* < 0.05). The cutoff values of PI and RI differentiating the women with URPL from the controls were confirmed by ROC and Youden’s index. Given a PI cutoff value of 2.6, the prevalence of URPL was significantly elevated in the high-PI group (74.58%) compared with that in the low-PI group (40.65%, *P* < 0.0001), with sensitivity and specificity of 63% and 69%, respectively. With an RI cutoff value of 0.86, the prevalence of URPL in the high-RI group (65.28%) was significantly elevated compared with that in the low-RI group (42.25%, *P* = 0.001), with sensitivity and specificity of 66% and 75%, respectively. The levels of serum D-dimers and anticardiolipin antibody (ACA)-IgM in URPL women were significantly higher than those in the controls. A positive correlation existed between the levels of ACA-IgM and uterine artery RI in URPL women (r = 0.43, *P* < 0.01). These results indicated that URPL women may be at a relatively high risk of a prothrombotic state, and the increased ACA-IgM deserves attention for its role in the elevated uterine artery Doppler parameters in URPL women.

## Introduction

Recurrent pregnancy loss (RPL) is described as two or more consecutive pregnancy losses that occur before 20–24 weeks of gestation. This condition affects 2%–5% of women attempting to conceive and poses a major concern for reproductive medicine manufacturers [1, 2]. Regardless of abnormalities, including genetic, anatomical, metabolic, and endocrinological factors, the prethrombotic state (PTS), and autoimmune diseases, explain only 50%–60% of the causes of RPL [3, 4]. Approximately 40%–50% of RPLs remain unexplained. Thus far, women with unexplained RPL (URPL) have been subjected to various empirical or nonevidence-based treatments with uncertain therapeutic effects and safety [2]. URPL treatment can be improved through further exploration of the etiology of URPL.

Uterine perfusion regulates endometrial receptivity, and impaired uterine perfusion may play a role in the pathogenesis of RPL, such as RPL associated with antiphospholipid syndrome [5]. However, the potential role of uterine arterial blood flow in URPL pathogenesis is still debated.

Pulsed Doppler ultrasonography of uterine arteries has been widely used in the evaluation of uterine blood perfusion in women with RPL. The increased resistance to uterine blood flow may be a crucial contributing factor to some causes of RPL and an independent indication of the risk of pregnancy loss [5, 6]. A study revealed significantly higher uterine artery pulsatility index (PI) values of RPL patients compared with those of the control group [7, 8]. However, published studies on the role of uterine perfusion in nonpregnant women with URPL are limited. Moreover, little is known regarding its potential influencing factors. This study was aimed at determining alterations in uterine artery Doppler parameters in nonpregnant women with URPL and exploration of the possibly associated serum markers.

## Methods

### Study Population

This retrospective case–control study was performed in West China Second Hospital of Sichuan University from Sep 2020 to Jan 2023 including 107 cases of women with URPL and 107 control women. The URPL group included women who had experienced two or more consecutive first trimester unexplained pregnancy losses. The control group included women who had at least one successful pregnancy with no history of first trimester pregnancy loss. Patients who had genetic anomalies, previous abortus’ chromosomal abnormality, thyroid abnormalities, PCOS associated with suspected luteal phase deficiency, inherited or acquired thrombophilia, antiphospholipid antibody syndrome, uterine anatomic abnormalities, uterine adhesion, and those with an autoimmune disease or abnormal blood pressure were excluded. Patients who received hormonal treatment 3 months before the study, smokers and those with chronic diseases or chronic ongoing treatments were also excluded as well. This study was approved by the Ethics Committee of West China Second Hospital.

### Doppler Measurement

Patients in both groups were scanned by transvaginal Doppler ultrasonography, using ultrasound equipment (GE Voluson E8) with a 6 MHz transvaginal transducer during the midluteal phase (6 to 8 days after ovulation) to measure the pulsatility index (PI), resistance index (RI) and systolic-to-diastolic ratio (S/D) values of the left and right main uterine. Doppler assessments were performed by operators with extensive experience in uterine artery flow assessment to minimize inter-observer variability. In this study, the mean values of PI, RI, and S/D of the right and left uterine arteries were selected as the indexes of uterine arterial blood flows.

### Detection for Immunologic and Hematological Parameters

Fasting peripheral blood samples were collected during the midluteal phase and for URPL women samples were collected at least 12 weeks after the last abortion. Serum anticardiolipin antibodies (ACA) –IgA, ACA-IgM and ACA-IgG were detected by the standard Cardiolipin-IgA, Cardiolipin-IgM and Cardiolipin-IgG kits (HOB Biotech Group Corp, Suzhou, China) and chemiluminescent microparticle immunoassay on the Bio-CLIA 6,500 automatic chemiluminescent immunoassay analyzer (Chongqing Keysmile Biological Technology Co, Chongqing, China). Serum D-dimer was measured using immunoturbidimetric assay. Total blood count parameters, including platelet count (PLT), platelet distribution width (PDW), mean platelet volume (MPV) and plateletcrit (PCT) were detected by Automated Haematology Analyser XN9000.

### Statistical Analysis

Data were expressed as mean ± standard deviation. Data were analyzed using the SPSS version 26 software (IBM Corporation, Armonk, NY) and MedCalc® Statistical Software version 20.100 (MedCalc Software Ltd, Ostend, Belgium). Differences between two groups were analyzed using the Student’s t-test for independent samples if the samples presented a normal distribution, or by the Wilcoxon rank test otherwise. Correlation between two variables was performed using Pearson correlation analysis. The Chi-square statistic is used to analyze prevalence rate of URPL in population with different characteristics. The Receiver Operator Characteristic (ROC) curves were used to evaluate the predictive performance of uterine artery flow indexes and clinical outcome variables. The areas under the ROC curves (AUCs) were compared using the Z-test. *P*-values < 0.05 were considered statistically significant.

## Results

### Clinical Characteristics of the Study Population

This work revealed no significant differences between the two groups in terms of age, body mass index (calculated as weight in kilograms divided by the square of height in meters), serum anti-Mullerian hormone, and baseline endocrine data, including estradiol, follicle-stimulating hormone, luteinizing hormone, and testosterone (*P* > 0.05; Table 1).

**Table 1:** Baseline characteristics of studied population

Variables	URPL group (*n* = 107)	Control group (*n* = 107)	*P* value
Age(years)	31.13 ± 3.34	31.52 ± 3.82	0.08
BMI(kg/m^2^)	22.09 ± 2.11	21.71 ± 2.41	0.32
AMH (ng/ml)	3.90 ± 3.18	3.71 ± 3.01	0.25
bE_2_ (pg/ml)	59.05 ± 34.13	58.87 ± 31.33	0.16
bFSH (IU/L)	6.39 ± 3.17	6.71 ± 2.82	0.13
bLH (IU/L)	5.23 ± 3.20	5.37 ± 3.59	0.32
Testerone (ng/ml)	0.29 ± 0.22	0.28 ± 0.16	0.29

Data are presented as mean ± SD

*URPL* unexplained recurrent pregnancy loss, *BMI* body mass index, *AMH* antimullerian hormone, *bE2* basal estrodial, *bFSH* basal follicle stimulating hormone, *bLH* basal luteinizing hormone

*P* value < 0.05 was regarded as statistically significant

### Comparison of Uterine Arterial Blood Flow Indexes Between URPL Women and Controls

Doppler assessment of uterine blood flow indicated the significantly higher midluteal phase PI, RI, and S/D for uterine arterial blood flow in women with URPL than those in the controls (Table 2).

**Table 2:** Comparison of Doppler parameters of uterine artery in the URPL group and control group

Variable	URPL group (*n* = 107)	Control group (*n* = 107)	*P* value
PI	2.49 ± 0.59	2.21 ± 0.38	< 0.001
RI	0.84 ± 0.05	0.82 ± 0.05	< 0.001
S/D	6.79 ± 2.17	6.22 ± 1.77	0.017

Data are presented as Mean ± SD

*URPL* unexplained recurrent pregnancy loss, *PI* pulsatility index, *RI* resistance index, *S/D* systolic-to-diastolic ratio

*P* value < 0.05 was regarded as statistically significant

### Association of Uterine Arterial Blood Flow Indexes with URPL

**Fig. 1:**
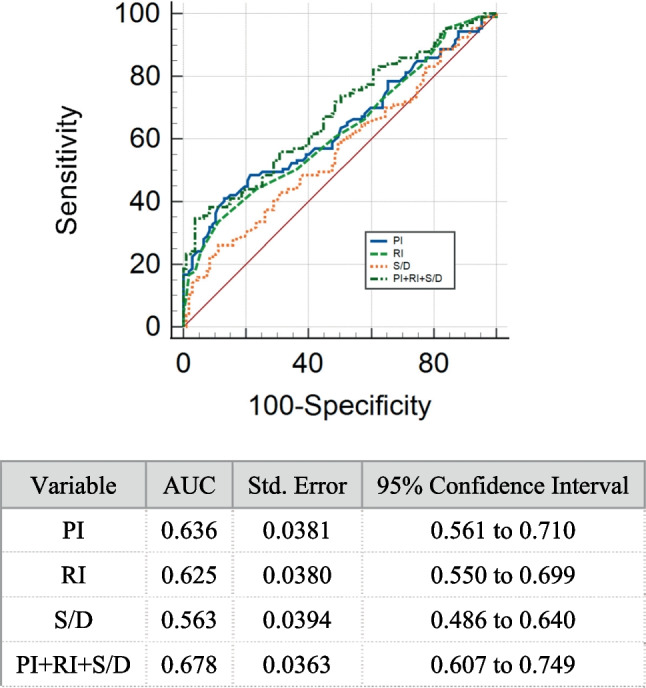
The ROC curve of uterine artery blood flow indexes for predicting URPL. ROC, receiver-operating characteristic; AUC, area under the curve; PI, pulsatility index; RI, resistive index; S/D, systolic-to-diastolic values

In the prediction of URPL, the mean PI, RI, and S/D had areas under the receiver operating characteristic (ROC) curve (AUCs) equal to 0.635, 0.625, and 0.563, respectively. S/D exhibited a significantly lower AUC than the combination of PR, RI, and S/D (0.678, Z = 2.752, *P* = 0.0059) (Fig. 1). No differences existed between the AUC of PI and the combination of PR, RI, and S/D and between those of the RI and the combination of PR, RI, and S/D. These findings imply the comparable prediction effect of PI or RI alone to that of combined PI, RI, and S/D.

At the midluteal phase, the cutoff values of PI, RI, and S/D differentiating the women with URPL from the controls were 2.60, 0.86, and 7.79, respectively, which were determined using ROC and Youden’s index (Table 3). At the PI cutoff value of 2.6, the sensitivity and specificity reached 63% and 69%, respectively. Given a PI cutoff value of 2.6, a total of 59 and 155 subjects were categorized into the high- and low-PI groups, respectively. The high-PI group exhibited a significantly higher prevalence of URPL (74.58%) than the low-PI group (40.65%, x^2^ = 19.68, *P* < 0.0001). The positive predictive and negative values of PI in the prediction of URPL were 75.48% and 59.35%, respectively. The cutoff value of RI (0.86) revealed sensitivity and specificity of 66% and 75%, respectively. At the RI cutoff value of 0.86, a total of 72 and 142 subjects were categorized into the high- and low-RI groups, respectively. The former presented a significantly higher prevalence of URPL (65.28%) than the low-RI group (42.25%, x^2^ = 10.131, P = 0.001). The positive predictive and negative values of RI in the prediction of URPL reached 65.28% and 57.75%, respectively.

**Table 3:** The predictive accuracies to predict women at high risk of URPL by the ROC analysis of uterine artery blood flow indexes

Parameters	Diagnostic threshold	Sensitivity (%)	Specificity (%)	Youden Index
PI	2.60	41.1	86.9	0.28
RI	0.86	33.6	88.8	0.224
S/D	7.79	26.2	88.8	0.15

*PI* pulsatility index, *RI* resistance index, *S/D* systolic-to-diastolic ratio, *ROC* receiver operating characteristics

*P* value < 0.05 was regarded as statistically significant

### Comparison of Immunologic and Hematological Parameters Between Control and URPL

Routine hematological parameters, including D-dimers, platelet indexes (such as PLT, PDW, MPV, and PCT), and common antibodies related to RPL (ACA, including ACA-IgA, ACA-IgM, and ACA-IgG), were analyzed. The control and URPL groups showed no significant differences in the levels of PLT, PDW, MPV, PCT, ACA-IgA, and ACA-IgG. The URPL women presented significantly higher D-dimer and ACA-IgM levels than the controls (Table 4).

**Table 4:** Blood parameters and serum ACA antibodies in the URPL group and control group

Variable	URPL group (*n* = 107)	Control group (*n* = 107)	*P* value
D-dimer (mg/L FEU)	0.49 ± 0.47	0.33 ± 0.29	< 0.001
ACA-IgA (APL/mL)	1.55 ± 1.41	1.54 ± 1.44	0.48
ACA-IgM (MPL/mL)	7.36 ± 4.94	5.21 ± 3.72	< 0.001
ACA-IgG (GPL/mL)	1.39 ± 1.29	1.44 ± 1.23	0.39
PLT (10^9/L)	214. ± 58.76	222.34 ± 86.98	0.12
PDW (fL)	14.32 ± 3.83	13.96 ± 3.84	0.22
MPV (fL)	11.47 ± 1.85	11.31 ± 1.42	0.22
PCT (%)	0.24 ± 0.05	0.25 ± 0.07	0.13

Data are presented as mean ± SD

*URPL* unexplained recurrent pregnancy loss, *ACA* anticardiolipin antibody, *Ig* immune globulin, *PLT* platelet count, *PDW* platelet distribution width, *MPV* mean platelet volume, *PCT* plateletcrit

*P* value < 0.05 was regarded as statistically significant

### Correlation of Serum D-dimers with Uterine Arterial Blood Flow Indexes

**Fig. 2:**
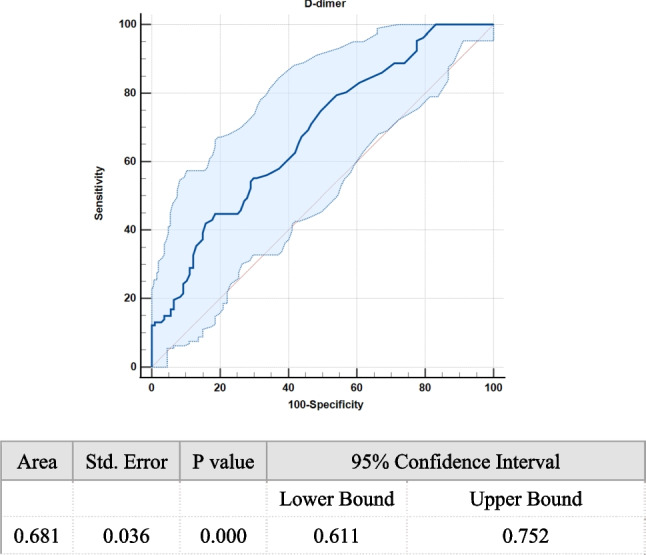
The ROC curve of D-dimer for predicting URPL. ROC, receiver-operating characteristic; AUC, area under the curve

**Fig. 3:**
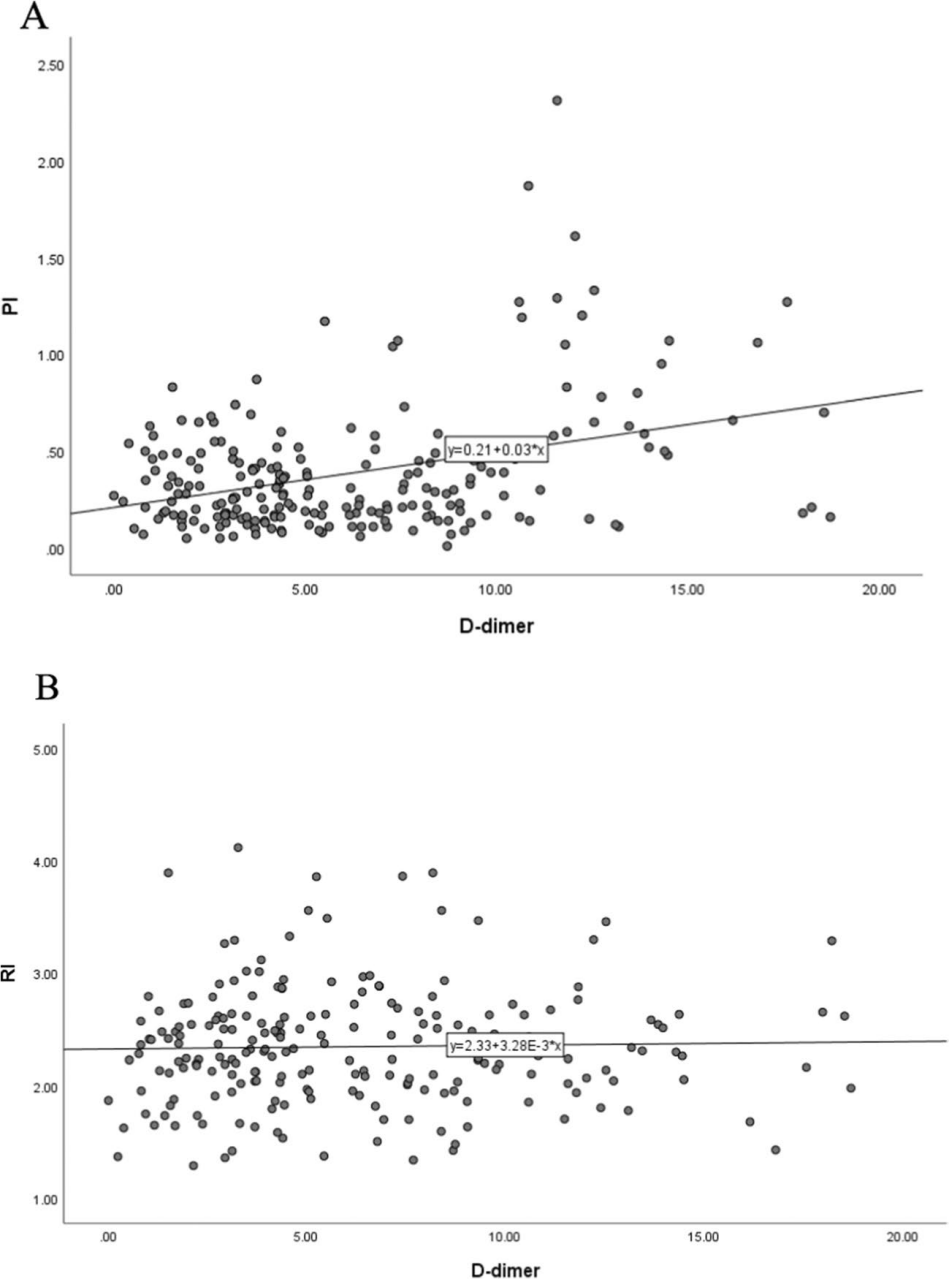
Correlation between the level of D-dimer and uterine artery PI or RI in the control and URPL groups. **A** The correlation between the level of D-dimer and uterine artery PI. **B** The correlation between the level of D-dimer and uterine artery RI. PI, pulsatility index; RI, resistive index

According to the ROC curve analysis, the AUC of D-dimer in the prediction of URPL was 0.681 (Fig. 2). A cutoff value of 0.435 was obtained, with a sensitivity ratio of 44.9% and a specificity ratio of 81.3%. At a cutoff value of 0.435 for the D-dimer, 69 and 145 subjects were categorized into the high- and low-D-dimer groups, respectively. The high-D-dimer group showed a significantly higher prevalence of URPL (71.01%) than the low-D-dimer group (40.0%, x^2^ = 21.598, *P* < 0.0001). The positive and negative predictive values of the D-dimer in the prediction of URPL were 71.01% and 60%, respectively. The correlation analysis showed no statistically significant positive correlation between the levels of D-dimers and uterine artery PI (r = 0.039, *P* = 0.571) or RI (r = 0.023, *P* = 0.736) in the control and URPL groups ( Fig. 3A and B).

### Correlation of ACA-IgM Levels with Uterine Arterial Blood Flow Indexes

**Fig. 4:**
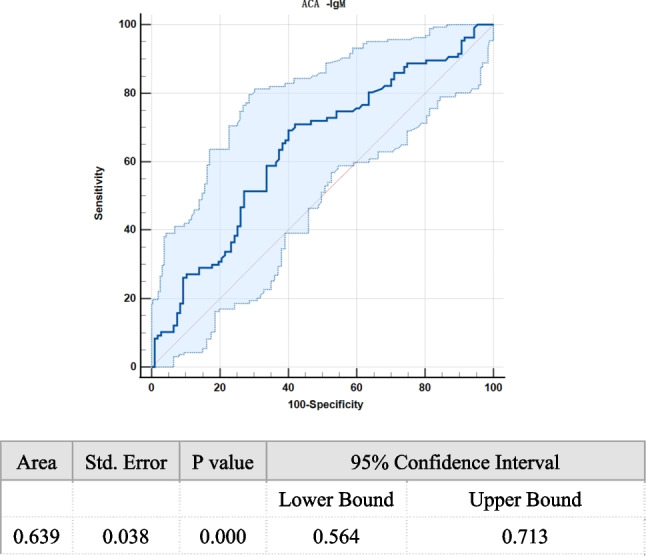
The ROC curve of ACA-IgM for predicting URPL. ROC, receiver-operating characteristic; AUC, area under the curve; ACA, anticardiolipin antibody; Ig, immune globulin

The ROC curve analysis revealed a 0.639 AUC of ACA-IgM in the prediction of URPL (Fig. 4). For ACA-IgM differentiating the women with URPL from the controls, the cutoff point was 4.41 with a sensitivity ratio of 69.2% and a specificity ratio of 59.8%. Based on the cutoff value of 4.41 for ACA-IgM, 117 and 97 subjects were categorized into the low- and high-ACA-IgM groups, respectively. The high-ACA-IgM group showed a significantly higher prevalence of URPL (63.25%) than the low-ACA-IgM group (34.02%, x^2^ = 18.121, *P* < 0.0001). The positive and negative predictive values of ACA-IgM in the prediction of URPL were 65.98% and 60%, respectively.

**Fig. 5:**
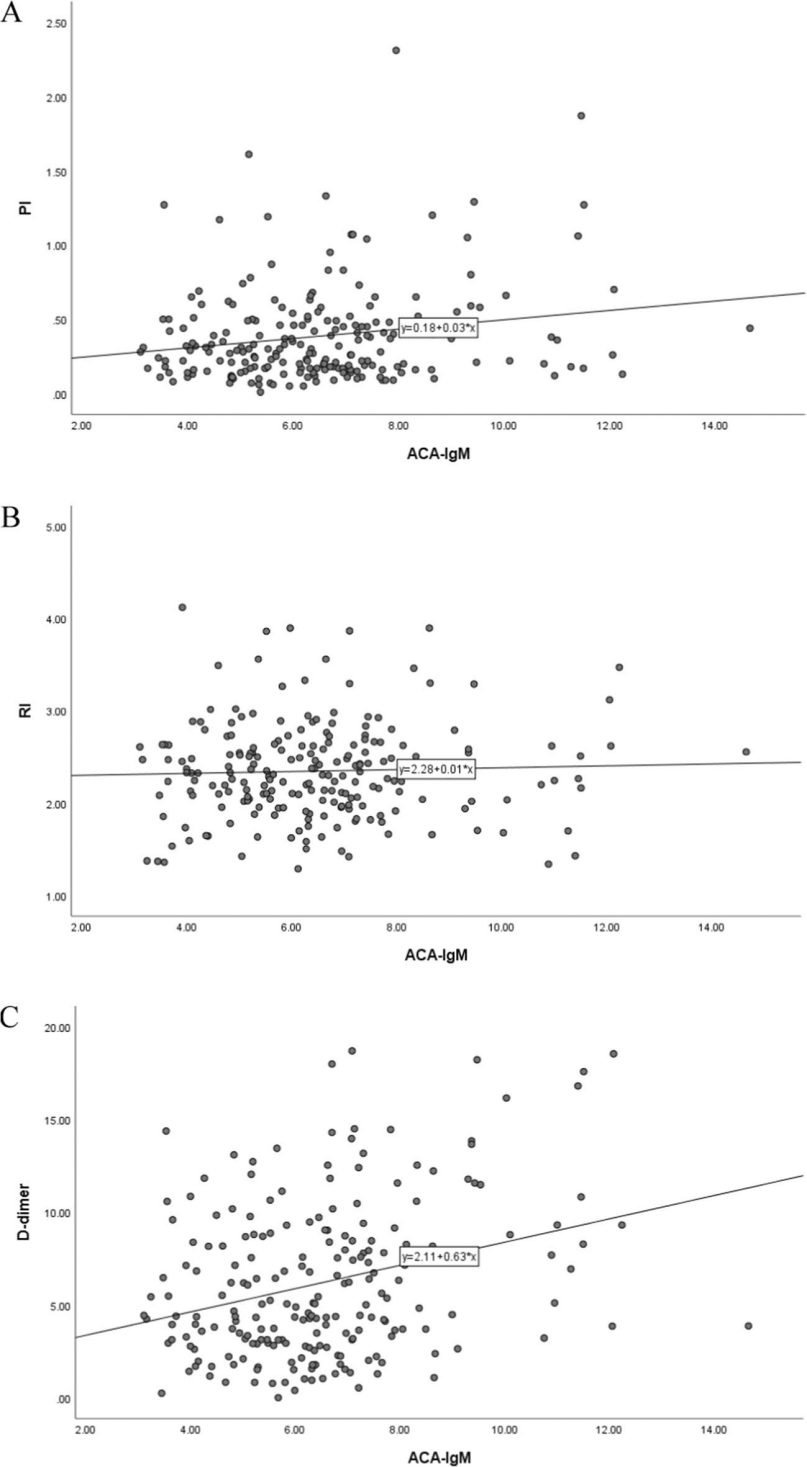
Correlation between the level of ACA-IgM and uterine artery PI, RI or D-dimer in the control and URPL groups. **A** The correlation between the level of ACA-IgM and uterine artery PI. **B** The correlation between the level of ACA-IgM and uterine artery RI. **C** The correlation between the level of ACA-IgM and D-dimer. ACA, anticardiolipin antibody; Ig, immune globulin; PI, pulsatility index; RI, resistive index

A statistically significant positive correlation was found between ACA-IgM and RI (r = 0.43, *P* < 0.01) in the URPL women. On the contrary, the control and URPL groups revealed no statistically significant correlation between the levels of ACA-IgM and uterine artery PI (r = 0.026, P = 0.703) or between those of ACA-IgM and serum D-dimers (r = 0.007, *P* = 0.915) (Figs. 5A–5C).

### Predictive Values of Single and Combined Detection of ACA-IgM, D-dimer, PI, RI, and S/D for URPL

**Fig. 6:**
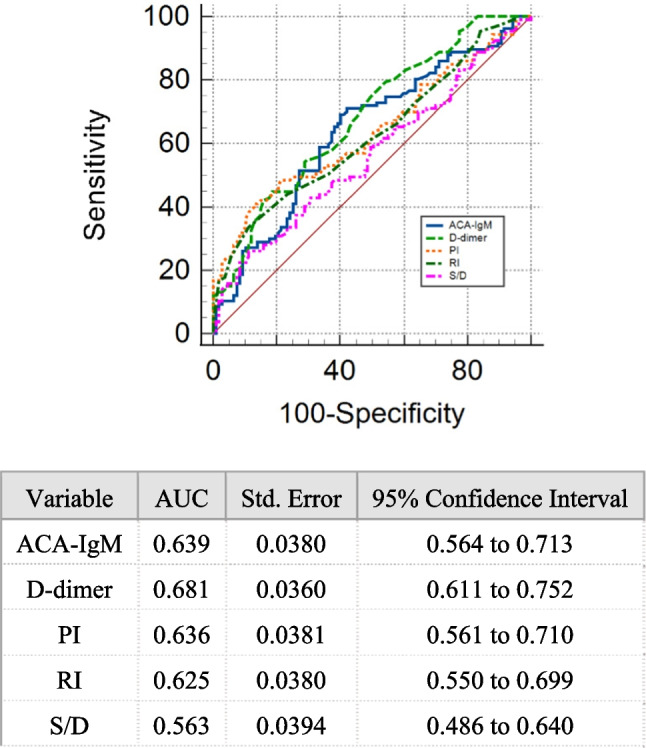
The ROC curve of ACA-IgM, D-dimer, PI, RI and S/D for predicting URPL. ROC, receiver operating characteristic; AUC, area under the curve; ACA, anticardiolipin antibody; Ig, immune globulin; PI, pulsatility index; RI, resistive index; S/D, systolic-to-diastolic values

The ROC curves were compared to evaluate the discrimination performance of single or combined detection of ACA-IgM, D-dimer, PI, and RI. The S/D attained a significantly lower AUC than the D-dimer (z = 2.32, *P* = 0.0203, 95% confidence interval (CI): 0.0184–0.218). No significant differences existed among the AUCs of PI, RI, and D-dimer (Fig. 6).

**Fig. 7:**
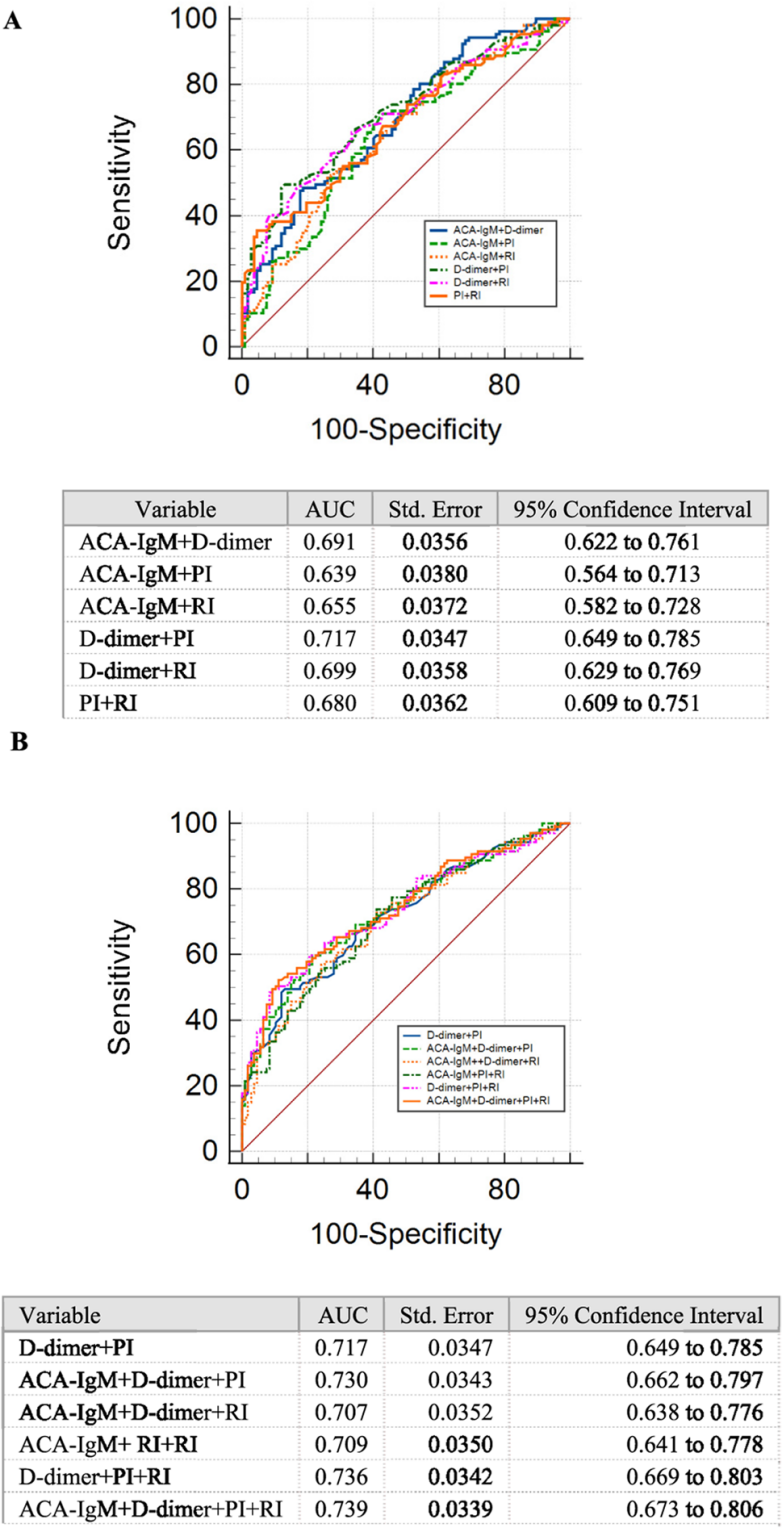
ROC of combined detection of ACA-IgM, D-dimer, PI and RI for predicting URPL. **A** Comparison of ROC of two-joint-detection of ACA-IgM, D-dimer, PI and RI for predicting URPL. **B** Comparison of ROC of D-dimer plus RI with three-joint or four-joint detection of ACA-IgM, D-dimer, PI and RI for predicting URPL. ROC, receiver operating characteristic; AUC, area under the curve; ACA, anticardiolipin antibody; Ig, immune globulin; PI, pulsatility index; RI, resistive index

Two-joint-parameter detection revealed the excellent predictive performance of the combined D-dimer and PI (AUC = 0.717, standard error (SE) = 0.0347, 95% CI: 0.649–0.785), and it was comparable to that of the three- or four-joint-parameter detection according to the ROC curve analysis. No differences existed among the AUCs of three-joint-parameter detection (Figs. 7A and B).

## Discussion

Successful pregnancy and reproduction show dependency on adequate uterine blood flow, placental perfusion, and vascular responsivity to fetal demands [9]. The midluteal phase involves the progressively diminishing impedance to uterine arterial blood flow as it reaches the lowest values, whereas the luteal phase includes progressive increase in the uterine arterial blood flow as it reaches the highest flow. Both scenarios are encountered in the period that temporally coincides with the implantation window; notably, uterine arterial blood flow parameters can be effective indexes for the evaluation of endometrial receptivity [10, 11]. Impaired uterine perfusion may be related to vascular damage, induce low endometrial receptivity, and contribute to the pathogenesis of RPL [12]. Some studies indicated that increased resistance to uterine blood flow may be a crucial contributing factor to some causes of RPL [13, 14]. However, the possible association of uterine arterial blood flow indexes with URPL remains unclear.

In this study, we evaluated the uterine artery flow indexes in women with and without URPL during the midluteal phase of untreated nonconception cycles. We observed that uterine artery PI increased in the URPL women compared with that in the controls, consistent with previous findings [5, 8]. In addition to PI, the RI and S/D were significantly higher in women with URPL than in women without URPL. The PI, RI, and S/D leading to the maximum values of sensitivity were 2.60, 0.86, and 7.79, respectively, which differentiated the women with URPL from the controls. A significantly higher prevalence of URPL was observed in women with uterine arterial blood flow indexes above the cutoff value, and it indicates the feasibility of using the uterine arterial blood flow indexes determined by Doppler in the midluteal phase of nonpregnancy cycles in the prediction of the risk of URPL. However, the causes of differences in the uterine artery flow indexes in URPL women remain poorly understood.

Therapies for URPL are currently empirical or experimental for the unclarified etiology, and their effectiveness have been controversial. Although several studies reported the improved pregnancy outcomes using anticoagulation therapy with aspirin or low-molecular-weight heparin in URPL women [7, 15–17], the underlying mechanism is unclear. Certain patients with URPL may have certain degree of hypercoagulability, although their condition fails to meet the diagnostic criteria for PTS. Given this assumption, routine hematological indexes, including platelet parameters and D-dimer (one of the most commonly used indicators for PTS), were analyzed in this study to determine their relation to URPL. An increase in PDW has been observed in women with URPL, and it was assumed to be associated with the high odds of URPL [18]. A preliminary study indicated the significantly higher MPV levels in URPL women [19]. No differences in the platelet indexes, including PDW, MPV, PLT and PCT, were observed between the URPL women and controls in this study. More studies are needed to determine the predictive value of platelet parameters in URPLprediction. Consistent with a previous study [20], we observed that the serum level of D-dimers in the URPL women was significantly higher than that in the controls, which suggests the presence of a relatively hypercoagulable state in the URPL women. The cutoff value of D-dimer was 0.435 mg/L FEU, as indicated by the ROC and Youden’s index, with women with D-dimer levels above the cutoff value revealing high prevalence rates of URPL. D-dimer may be regarded as a potentially predictive indicator for URPL.

The combination of ACA and vascular endothelial phosphatide can cause damage to the vascular endothelium and local thrombus formation, which result in insufficient blood supply to the decidual membrane and placenta, vascular lesions, placental embolism, and infarction [21, 22]. ACA also undergoes a reaction with platelets or the membrane phospholipids of vascular endothelial cells [23]. As a result, local blood vessel contraction, platelet aggregation, and a decrease in the blood volume of the placenta occur, which leads to pathological pregnancy [24, 25]. This study excluded ACA-positive women. Although at a negative level, significantly higher ACA-IgM values were observed for the URPL women in this study compared with the controls. Significantly high prevalence rates of URPL were detected in women with an ACA level above the cutoff value of 4.41. The correlation analysis revealed the positive correlation of ACA-IgM with RI. The elevated levels of ACA-IgM in the URPL women may increase hypercoagulability, which manifested as elevated levels of D-dimers, through the destruction of endothelial cells. We speculate that this phenomenon contributes to impaired uterine perfusion and increased risk of miscarriage. These findings provide an empirical basis for the application of anticoagulation therapy in URPL women to a certain extent.

According to the ROC curve analysis, the predictive performance of S/D for uterine artery flow (AUC = 0.563) was considerably lower than that of D-dimer (AUC = 0.681). The two-joint-parameter detection exhibited an excellent predictive performance for the combined D-dimer and PI (AUC = 0.717, SE = 0.0347, 95% CI: 0.649–0.785), combined D-dimer and RI (AUC = 0.699, SE = 0.0358, 95% CI: 0.629–0.769), and combined ACA-IgM and D-dimer (AUC = 0.691, SE = 0.0356, 95% CI: 0.622–0.761). No significant differences were found in the predictive performance among the detection of two joint parameters. The ROC curve analysis revealed the comparable predictive performance of the combined detection of D-dimer with PI, D-dimer with RI, and ACA-IgM with D-dimer to that of the detection of three joint parameters. No differences existed among the AUCs of the three-joint-parameter detection. Therefore, the combined use of D-dimer and PI, D-dimer and RI, or the combined detection of ACA-IgM and D-dimer has almost equal performance with the detection of three joint indexes in URPL prediction.

In conclusion, this study demonstrated that compared with normal controls, a high proportion of women with URPL possibly had impaired uterine perfusion and a potentially relatively high risk of experiencing a PTS. Potentially predictive indicators for URPL includes a midluteal phase Doppler assessment of the uterine artery PI and RI and detection of serum ACA-IgM and D-dimer, which may be possibly used as cost-effective, noninvasive means of etiological screening for URPL. The elevated ACA-IgM level showed a positive correlation with the RI increased uterine artery in URPL women and thus deserves research attention. Large-scale studies are also needed to verify the findings and clarify the possible etiology of URPL.

## Data Availability

The data are available from the corresponding author upon reasonable request.
